# A New Kind of Quinonic-Antibiotic Useful Against Multidrug-Resistant *S. aureus* and *E. faecium* Infections

**DOI:** 10.3390/molecules23071776

**Published:** 2018-07-19

**Authors:** Javier Campanini-Salinas, Juan Andrades-Lagos, Gerardo Gonzalez Rocha, Duane Choquesillo-Lazarte, Soledad Bollo Dragnic, Mario Faúndez, Pedro Alarcón, Francisco Silva, Roberto Vidal, Edison Salas-Huenuleo, Marcelo Kogan, Jaime Mella, Gonzalo Recabarren Gajardo, David Vásquez-Velásquez

**Affiliations:** 1 Drug Development Laboratory, Faculty of Chemical and Pharmaceutical, Sciences, Universidad de Chile, Sergio Livingstone 1007, Santiago 8380492, Chile; javier.campanini@uss.cl (J.C.-S.); jandrades@ug.uchile.cl (J.A.-L.); 2 Facultad de Medicina y Ciencia, Universidad San Sebastián, Lago, Panguipulli 1390, Puerto Montt 5501842, Chile; 3 Laboratorio de Investigación en Agentes Antibacterianos (LIAA), Departamento de Microbiología, Facultad de Ciencias Biológicas, Universidad de Concepción, Concepción 4070386, Chile; ggonzal@udec.cl; 4 Laboratorio de Estudios Cristalográficos, IACT (CSIC-UGR), Av. de las Palmeras 4, 18100 Armilla (Granada), Spain; duane.choquesillo@csic.es; 5 Bioelectrochemistry Laboratory, Faculty of Chemical and Pharmaceutical, Sciences, Universidad de Chile, Santiago 8380492, Chile; sbollo@ciq.uchile.cl; 6 Molecular Pharmacology and Toxicology Laboratory, Pharmacy Department, Faculty of Chemistry, Pontificia Universidad Católica de Chile; Santiago 7820436, Chile; mfaundeza@uc.cl; 7 Agents of bacterial meningitis laboratory, Instituto de Salud Pública de Chile, Santiago 7780050, Chile; palarcon@ispch.cl; 8 Microbiology Unit, Clinical Laboratory, Clinical Hospital University of Chile; Santiago 8380456, Chile; fsilva@hcuch.cl; 9 Antibiotics Laboratory, Microbiology Program, Biomedical Sciences Institute, Faculty of Medicine, Universidad de Chile, Santiago 8380453, Chile; rvidal@uchile.cl; 10 Nanobiotechnology and Nanotoxicology Laboratory, Faculty of Chemical and Pharmaceutical Sciences, Universidad de Chile, Santiago 8380494, Chile; edison.salash@gmail.com (E.S.-H.); mkogan@ciq.uchile.cl (M.K.); 11 Institute of Chemistry and Biochemistry, Faculty of Sciences, Universidad de Valparaíso, Playa Ancha, Valparaíso 2360102, Chile; jaime.mella@uv.cl; 12 Escuela de Quimica y Farmacia, Facultad de Medicina, Universidad Andres Bello, Quillota 980, Viña del Mar 2531015, Chile; 13 Laboratory of Synthesis of Bioactive Heterocycles, Pharmacy Department, Faculty of Chemistry, Pontificia Universidad Católica de Chile; Santiago 7820436, Chile; grecabarren@uc.cl

**Keywords:** quinonic-antibiotics, methicillin-resistant *Staphylococcus aureus*, vancomycin-resistant *Enterococcus faecium*, antibacterial activity

## Abstract

A rapid emergence of resistant bacteria is occurring worldwide, endangering the efficacy of antibiotics and reducing the therapeutic arsenal available for treatment of infectious diseases. In the present study, we developed a new class of compounds with antibacterial activity obtained by a simple, two step synthesis and screened the products for in vitro antibacterial activity against ATCC^®^ strains using the broth microdilution method. The compounds exhibited minimum inhibitory concentrations (MIC) of 1–32 μg/mL against Gram-positive ATCC^®^ strains. The structure–activity relationship indicated that the thiophenol ring is essential for antibacterial activity and the substituents on the thiophenol ring module, for antibacterial activity. The most promising compounds detected by screening were tested against methicillin-resistant *Staphylococcus aureus* (MRSA) and vancomycin-resistant *Enterococcus faecium* (VREF) clinical isolates. We found remarkable activity against VREF for compounds **7** and **16**, were the MIC_50/90_ were 2/4 µg/mL and 4/4 µg/mL, respectively, while for vancomycin the MIC_50/90_ was 256/512 µg/mL. Neither compound affected cell viability in any of the mammalian cell lines at any of the concentrations tested. These in vitro data show that compounds **7** and **16** have an interesting potential to be developed as new antibacterial drugs against infections caused by VREF.

## 1. Introduction

Infectious diseases are a leading cause of death worldwide, and the increasing emergence of antibacterial resistance has contributed to rising rates of potentially fatal infections. It is estimated that by 2050, diseases caused by antibiotic-resistant microorganisms will be responsible for 10 million deaths per year [1]. The bacteria implicated in these infections include the so-called ESKAPE pathogens, such as *Enterococcus faecium*, *Staphylococcus aureus*, *Klebsiella pneumoniae*, *Acinetobacter baumannii*, *Pseudomonas aeruginosa*, and various *Enterobacter* sp. [2,3]. These microorganisms have become a global public health problem due to their ability to adapt to antibacterial agents. *S. aureus*-related skin infections, wound infections, and sepsis cases are the leading causes of healthcare-associated infections [4], and about 90% of *S. aureus* isolates were found to be methicillin-resistant in a study using data from 21 of the 35 countries in the Americas [4]. In the United States (US), these resistant strains are responsible for over 11,000 deaths per year [5]. In addition, *Enterococcus* species cause significant numbers of urinary tract, surgical site, and blood infections [4]. In the United Kingdom [6] and the US [7], up to 25% and 60% of *E. faecium* strains are resistant to vancomycin (VAN), respectively.

Traditionally, development of new antibacterial molecules has been based mainly on two strategies: modifying or adding a small chemical group to an antibiotic already in clinical use, to improve some aspect of its pharmacodynamic and/or pharmacokinetic profile; or seeking new molecules from natural products such as plants, bacteria, or fungi that have demonstrated activity against resistant bacteria. Both strategies involve structural modifications or additions that preserve the pharmacophore and therefore maintain both the mechanism and site of action. Optimizing these compounds may be initially effective, however, due to the structural similarity between novel and existing molecules, bacteria rapidly adapt their resistance mechanisms to thwart new antibiotics [8]. The traditional chemical approach is effective for identifying and optimizing compounds to treat pathologies such as hypertension, diabetes, dyslipidemia, inflammation, and allergies, in which the pharmacological targets do not adapt or generate resistance mechanisms. Infections, however, pose a different challenge, as bacteria are free-living organisms that seek to survive in the presence a harmful agent.

If we continue to rely exclusively on these traditional strategies, it is only a matter of time before our entire investment in the generation of antibiotics is overwhelmed by antibacterial resistance. The way forward should focus on rational, design-oriented development of new synthetic molecules capable of reducing the probability that exposed bacteria will generate a resistance phenotype. Antibacterial agents that have novel chemical structures and that act on unexplored bacterial targets are less likely to be subject to existing compound- or target-based resistance mechanisms. Of course, even new classes of antibiotics may be subject to general mechanisms of resistance, such as increased efflux, reduced influx, or target-site resistance mutations [8].

New approaches should consider a target that is different from existing targets, essential for microbial cell survival, highly conserved in clinically relevant species, absent or radically different in human cells, and easy to assay and approach biochemically [9,10]. However, structural modifications based on the traditional medicinal chemistry approach will also be needed to optimize effectiveness, and rational design will require synthesis of multiple compounds in order to determine the relationship between structure and activity. Therefore, it is critical to use a simple, versatile, and low-cost process to synthesize these molecules.

In this regard, various activities have been attributed to quinonic compounds. These compounds present two important characteristics for drug design. First is the versatility of synthetic processes, which allows active compounds to be obtained in a few stages; the second is the broad spectrum of biological activities described, which shows that the choice of substituents is critical to guiding the objective of biological activity, as shown by the work of Gordaliza et al. [11]. Quinonic compounds exert interesting antibacterial effects [12,13,14] and have already been integrated into antibacterial compounds, such as alkannin [15] and renierone [16]. These quinone-based antibiotics have been found to have activity against *S. aureus*, *E. faecium*, and *Bacillus subtilis* [17,18]. Promisingly, Tandon et al. have shown that thioaryl substitution in naphtoquinone results in good antibacterial activity [19,20]. However, it has not been further studied how the substituents of quinone compounds are related to their antibacterial activity. This information can be used to guide the rational design of new antibacterial quinone compounds.

In this study, our group develop a new kind of antibacterial agent based structurally on the quinonic core. We synthesized and assessed a set of 17 compounds against American Type Culture Collection (ATCC^®^) bacterial strains and addressed the study of the structure–activity relationship based on lipophilicity (logP), half wave potential (E_1/2_), and volume (MR) parameters. We also analyzed the crystallographic structure of the two compounds with the best antibacterial performance and tested their activity against multidrug-resistant clinical isolates, to calculate minimum inhibitory concentration (MIC)_50/90_ and minimum bactericidal concentration (MBC)_50/90_ values against *S. aureus* and *E. faecium*. Finally, the toxicity of these two compounds against the HeLa, HTC-116, SHSY-5Y, and Vero cell lines was evaluated to assess their safety and suitability for use as a treatment.

## 2. Results and Discussion

### 2.1. Synthesis and Determination of Physicochemical Parameters

**Figure 1:**
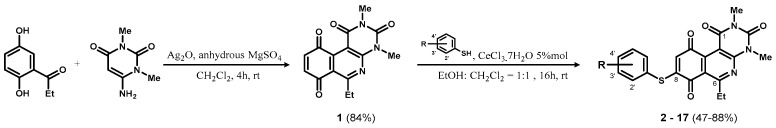
Synthetic route of the preparation of core **1** and compounds **2**–**17**.

The final compounds were formed in two steps. First, we used activated benzoquinone, generated in situ from the corresponding hydroquinone with silver (I) oxide, reacting with enaminone through an ionic [3+3] process to produce the corresponding core **1** with a yield of 84% by a further in situ oxidation. During the second step, compounds **2**–**17** were obtained by nucleophilic addition of the corresponding thiophenol derivatives to core **1** with subsequent aerobic oxidation of the hydroquinone formed by Michael addition (Figure 1). Yields ranged from 47% to 88% for the 2′-, 3′-, and 4′-series. The lipophilic (LogP), half-wave potential (E_1/2_) and molar refractivity (MR) parameters (Table 1) are discussed in Section 2.2. It should be noted that formation of the final products was highly favorable, with reactions produced under mild reaction conditions at room temperature. The total production time for each compound was 48 h. In addition, each product had a characteristic color that was distinct from that of the starting agent (yellow), facilitating purification by enabling rapid visual detection during the column chromatographic separation.

### 2.2. Antibacterial Screening

The antibacterial profile shows that all compounds except **5** and **6** demonstrated activity against Gram-positive bacteria (methicillin-resistant *S. aureus* [MRSA] ATCC^®^ 43300, methicillin-susceptible *S. aureus* [MSSA] ATCC^®^ 29213, and *E. faecalis* ATCC^®^ 29212). No activity was observed against Gram-negative bacteria (*Escherichia coli* ATCC^®^ 25922 and *P. aeruginosa* ATCC^®^ 27853), even at the highest concentrations tested (32 μg/mL, maximum solubility for the compounds). The antibacterial screening results are presented in Table 1. However, preliminary tests showed that adding EDTA to compounds [21] may induce activity against Gram-negative bacteria (unpublished results), through two possible mechanisms: fluidizing the lipid membrane [21] to facilitate passage of the derivatives through the membrane; or enabling the compound to act as a siderophore, in which the compound takes advantage of the high affinity of the iron transport system to facilitate entry into the bacterium [22]. This result suggests that it is possible to achieve activity against Gram-negative bacteria using a strategy that enables penetration of the bacterial membrane.

### 2.3. Structure-Activity Relationship

Core **1** showed no antibacterial activity, but addition of the thiophenol ring resulted in measurable activity (compound **2**). This observation suggests that the aromatic system is essential to activity. Substituents at the 2′-, 3′-, and 4′-positions of the thiophenol ring gave rise to the 2′-, 3′-, and 4′-series, respectively.

The 2′-series was comprised of compounds **3** through **7**. Using the activity of compound **2** as a reference, compounds **4** and **7** exhibited the highest antibiotic activity within this series and possessed the bulkiest substituents in the series, the methoxy group and the bromine atom, with MR values of 7.24 and 9.06 cm^3^/mol, respectively. However, the presence of a methyl group in compound **3** was associated with reduced activity, resulting in MIC values of 32 μg/mL for MRSA and MSSA and >32 μg/mL for *E. faecalis*. Finally, fluorine and chlorine substituents in the *ortho* position, which produced compounds **5** and **6**, respectively, showed no antibacterial activity. It is interesting to note the steric effect of the substituents at the C-2′ position of the thiophenol ring; apparently, the atomic volume forces the structure to assume a conformation with a semi-perpendicular dihedral angle that may reduce the degrees of freedom of the structure. This observation suggests that bulky groups at C-2′ favor antibacterial activity.

Compounds with *meta* substitutions, the 3′-series (compounds **8** through **12**), showed MIC values of 2 to 32 μg/mL. Within this series, it was not possible to obtain a correlation that explained the results of biological activity obtained according to the LogP, E_1/2_, or MR molecular descriptors. However, the methyl, methoxy, and fluorine substituents (compounds **8**, **9**, and **10**, respectively) were observed to have the best profiles in Gram-positive bacteria. On the other hand, interestingly, the halogenated compounds (**11** and **12**) were more potent against resistant than sensitive strains, because these compounds showed MIC values of 2 μg/mL MRSA strains, whereas potency against MSSA strains was lower, at 32 μg/mL.

The 4′-series compounds (**13** through **17**) showed MIC values ranging from 4 to 16 μg/mL. Compound **16** showed the highest potency. Considering the reported relationships between the quinonic structures, their redox capacity, and their biological activity [24,25], we tackled the study of the 4′-series compounds, considering that the thiophenol substituents could modulate antibacterial activity through control of the descriptor E_1/2_.

**Figure 2:**
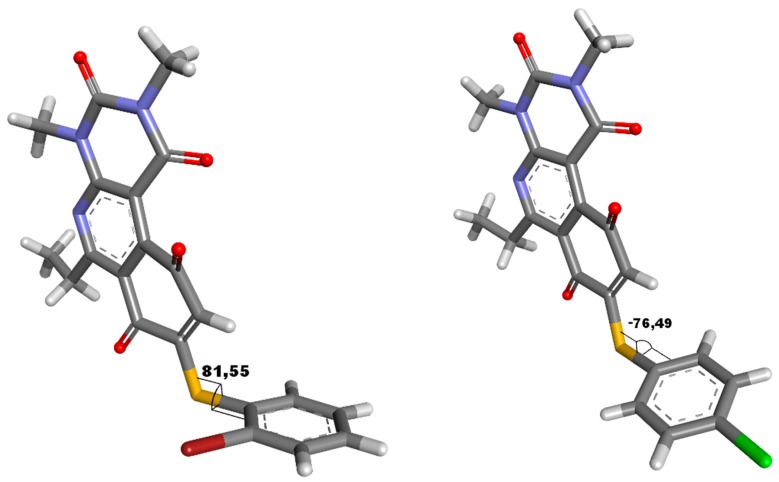
Dihedral angle for compounds **7** and **16**, determinate from structure crystalize resolute by X-ray diffraction.

The relationship established by Hammett between the type of substituent in the *para* position and reactivity is well known [26]. This analysis can be applied to the 4′-series, in that whether the substituent in position 4′ is an electron donor or acceptor will affect the quinonic nucleus through the sulfur atom, modifying the redox potential of the compounds. This effect requires that the aromatic systems, the thiophenolic ring and the quinonic nucleus, are in the same plane to maximize conjugation of the two systems, which can be extrapolated according to the value of the dihedral angle between the two aromatic systems. In order to determine the dihedral angle, compound **16** was crystallized and resolved using X-ray diffraction to produce a three-dimensional image of the structure. In this analysis, the most active compound of the 2′-series (compound **7**) was included to determine whether the insertion of a bulky substituent in position 2′ (bromine atom) significantly influences the dihedral angle. Images of the molecular structure showed that the dihedral angle between the aromatic ring and the quinone core was nearly perpendicular, with values of 81.6 and 76.5 degrees for compounds **7** and **16**, respectively (Figure 2).

These results demonstrate than the molecules do not present a dihedral angle sufficient to consider significant conjugation between the aromatic systems; therefore, the electronic effect of the substituent on the half-wave potential must be principally inductive. This observation is concordant with the narrow range of E_1/2_ values obtained for the compounds (−0.623 to −0.374 mV), suggesting that the inductive effect weakly affected the redox potential of the quinones. Interestingly, the bulky substituent in the *ortho* or *para* position did not significantly alter the dihedral angle, because the presence of the bromine atom in position 2′ increased the angle by 5 degrees as compared to molecules with a chloride atom in position 4′. This analysis for the 4′ series shows that the E_1/2_ parameter does not present a clear correlation with antibacterial activity. With respect to the lipophilicity parameter, no correlation was observed with activity in the 2′ series, since the compound with the lowest (compound **4**) and highest (compound **7**) logP showed similar activities in the series. For the 3′ series, despite the variation in lipophilicity, a narrow range of MIC values (2 to 8 ug/mL) was observed. Of the MSSA, the most lipophilic compounds of the 3′ series had the lowest activity. For the 4′ series, a linear correlation between logP and MIC was observed, where the compounds with the highest lipophilicity had the highest activities of the series.

In summary, the phenyl ring is essential for activity, the increased size of substituents in the *ortho* position improve antibacterial activity, and for the *meta* position, the physicochemical characteristics of the substituents do not show a correlation with antibacterial activity. In the *para* position, a lipophilic substituent improves antibacterial activity.

### 2.4. Antibacterial Activity of Compounds ***7*** and ***16*** Against Clinical MDR Isolates

To assess the clinical potential of the most promising compounds, we evaluated their antibacterial activity against heterogeneous populations of clinical isolates as well as their toxicity against human and animal cells. Compounds **7** and **16** were selected for this analysis, as these two compounds showed the greatest antibacterial activity.

**Figure 3:**
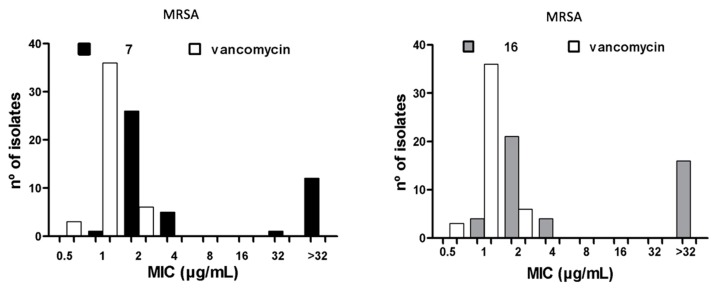

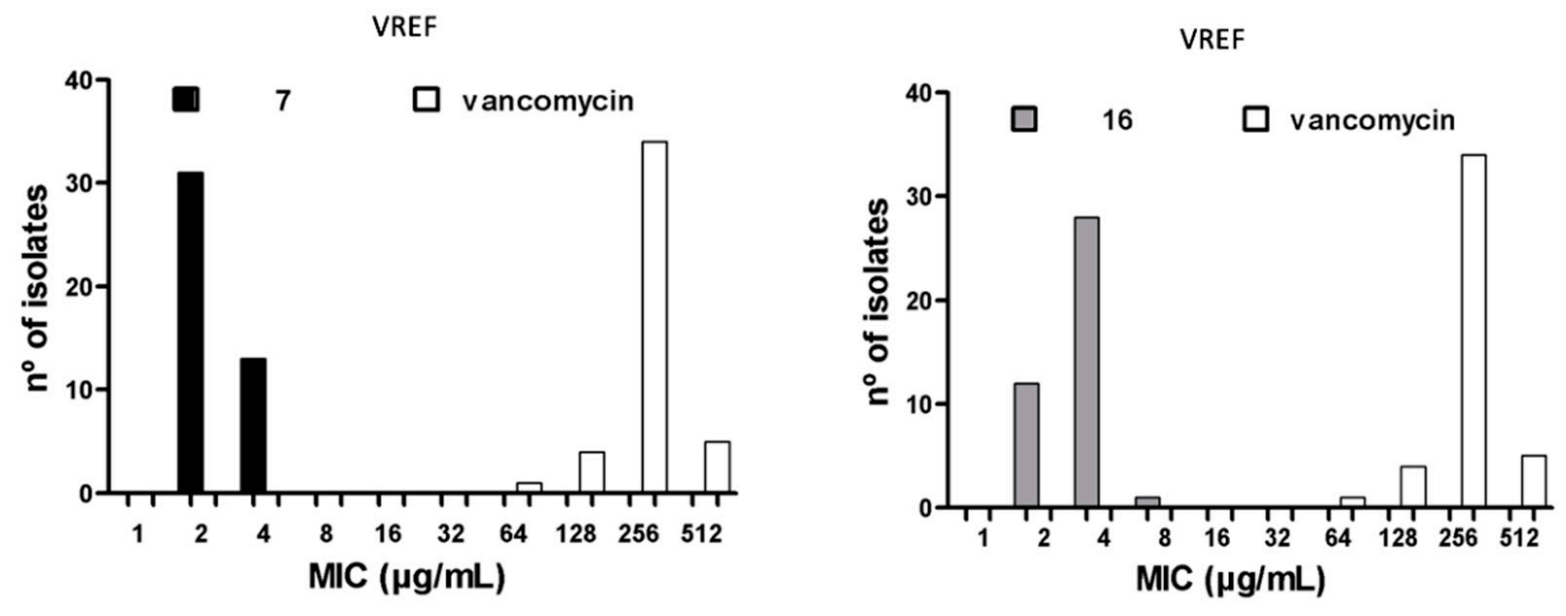
MIC_90_ histograms for compounds **7** and **16** against MRSA and VREF vs vancomycin.

The MIC_50/90_ and MBC_50/90_ against clinical isolates of MR**SA** and vancomycin-resistant *E. faecium* (VREF), as well as the geometric mean (MG) of the MIC parameters, were determined for compounds **7** and **16**. The MIC_50/90_ values reflect the effectiveness of antibacterial agents against a heterogeneous bacterial population. Vancomycin (VAN) and gentamicin (GEN) were used as quality controls; if the MIC values for these reference drugs against each bacterial strain were equal to the values reported by the Instituto de Salud Pública de Chile (ISP), the trials were considered valid. The maximum concentration tested was 32 μg/mL, as this value represents the solubility limit of the compounds. The results obtained for compounds **7**, **16**, and VAN against MRSA and VREF are summarized in Table 2 and Table 3, respectively, and the histograms are presented in Figure 3. For compound **7**, the MIC_50/90_ was 2/2 µg/mL, and the MBC_50/90_ was 4/4 µg/mL in 32 *S. aureus* isolates. For compound **16**, in contrast, the MIC_50/90_ was 2/4 µg/mL, and the MBC_50/90_ was 2/4 µg/mL in 29 *S. aureus* isolates. For VAN, the MIC_50/90_ was 1/1 µg/mL. The MBC_50/90_ was not assessed. For compound **7**, the MIC_50/90_ was 2/4 µg/mL, and the MBC_50/90_ was 4/4 µg/mL in 44 *E. faecium* isolates. For compound **16**, the MIC_50/90_ was 4/4 µg/mL and the MBC_50/90_ was 4/8 µg/mL in 41 *E. faecium* isolates. For VAN, the MIC_50/90_ was 256/512 µg/mL, and the MBC_50/90_ was not assessed.

### 2.5. Antibacterial Activity Against Heterogeneous Populations of Clinical Isolates

The bacterial population used to calculate the MIC_90_ values had a complex susceptibility profile, as most of the isolates tested were resistant to three or more classes of drugs. However, both compounds had MIC_90_ values near those of VAN against MRSA (MIC_90_ = 1 μg/mL, *p* > 0.001). Moreover, the two compounds had significantly lower MIC values than VAN against VREF isolates (64–256 μg/mL, *p* > 0.001). Compounds **7** and **16** were 128-fold and 64-fold more active than VAN, respectively, against the various clinical isolates of VREF. The MIC_90_ values for compounds **7** and **16** are similar to those reported for linezolid (2–4 μg/mL) against MRSA [27,28,29,30,31], showing that these molecules may offer potential therapeutic alternatives to VAN for the treatment of skin infections caused by MRSA [32]. Linezolid has been found to have MIC_90_ values of 2–64 μg/mL against *Enterococcus* spp. [27,29,33], indicating that linezolid is a therapeutic option for treating urinary tract infections caused by VREF. In addition, compounds **7** and **16** showed activity similar to or greater than VAN and linezolid against MRSA and multidrug-resistant *E*. *faecium* isolates. Furthermore, compounds **7** and **16** were found to have MBC values equal to or up to two times greater than the MIC values against all strains tested, including MRSA (32 isolates) and VREF (44 isolates). An MBC/MIC ratio of 2 or lower indicates that a compound has a bactericidal mode of action, according to Craig et al. [34], suggesting that these compounds were successful in killing the clinical isolates of MRSA and VREF.

### 2.6. Cytotoxicity of Compounds ***7*** and ***16***

**Figure 4:**
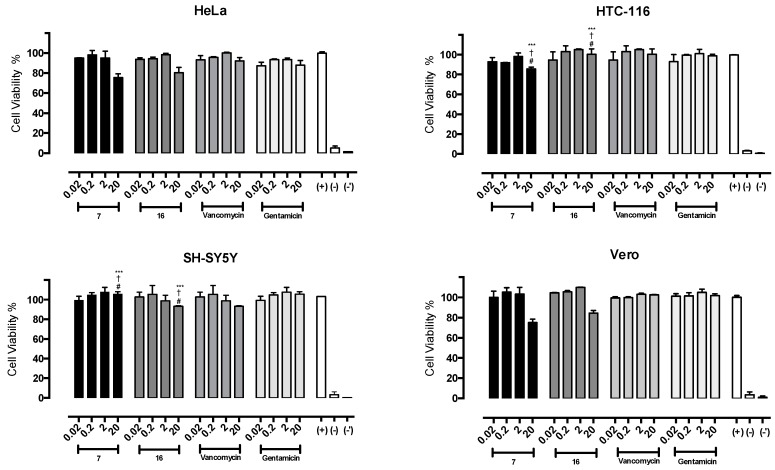
In vitro toxicity of compounds **7** and **16** (*n* = 3) over HeLa, HTC-116, SH-SY5Y and Vero cell lines, in the range of 0.02 to 20 µg/mL in triplicated. (+) Positive growth control, RPMI 140 medium. (−) Negative growth control. (−′) Negative growth additional control, 1% DMSO + H_2_O. Vancomycin and Gentamicin were used as drug reference. ***: *p* < 0.001 = statistically no significant differences with positive growth control; #: *p* < 0.001 = statistically no significant differences with vancomycin; †: *p* < 0.001 = statistically no significant differences with vancomycin.

To test the safety of compounds **7** and **16** in mammalian cells, cytotoxicity studies were performed using the HeLa, HTC-116, SH-SY5Y, and Vero cell lines. A colorimetric assay was performed to estimate the half-maximal inhibitory concentration (IC_50_) values, which represent the concentration of a drug that is required for 50% inhibition in vitro after 24 h of continuous exposure to the compound. Four serial dilutions (from 0.02 to 20 µg/mL) for each sample were evaluated in triplicate in three reproducible assays, and VAN and GEN were used as reference drugs. The results are shown in Figure 4.

It was not possible to determine the IC_50_ values because the viability percentages observed were greater than 75% at all concentrations studied. No significant differences between compounds **7** or **16** (*p* > 0.001) and the positive control (*p* > 0.001) or the reference drugs (*p* > 0.001) were observed.

The toxicity results for mammalian cells, including human (HeLa, HTC-116, and SHSY-5Y) and animal (Vero) cells, showed no significant differences between either of the two compounds and the positive control (*p* > 0.05), indicating that the compounds did not affect the viability of the cells after 24 h of exposure at any of the concentrations studied (0.002–20 μg/mL). Moreover, there were no significant differences in toxicity between the two novel compounds and the reference antibacterial agents (VAN and GEN) at any of the concentrations tested (*p* > 0.05). Due to the low solubility of the compounds, it was not possible to determine their IC_50_ in cell culture. At the maximum concentration tested, 70% cell viability was observed, a percentage that is insufficient for calculation of the IC_50_.

Finally, there was a wide gap between the antibiotic and toxic concentrations of the compounds. For example, it would be necessary to increase the concentration of compounds **7** and **16**, which showed MIC values of 2 and 4 μg/mL against MRSA, respectively, by a factor of 5 to 10 before the viability of mammalian cells (HeLa and Vero) would be affected.

## 3. Materials and Methods 

### 3.1. Materials

The compounds were synthesized from the commercial precursors 1-(2,5-dihydroxyphenyl)-propan-1-one, 6-amino-1,3-dimethyl-2,4(1*H*,3*H*)-pyrimidinedione, 2-bromobenzenethiol, 2-chloro-benzenethiol, 2-fluorobenzenethiol, 2-methoxybenzenethiol, 2-methylbenzenethiol, 3-bromobenzene-thiol, 3-chlorobenzenethiol, 3-fluorobenzenethiol, 3-methoxybenzenethiol, 3-methylbenzenethiol, 4-bromobenzenethiol, 4-chlorobenzenethiol, 4-fluorobenzenethiol, 4-methoxybenzenethiol, and 4-methyl-benzenethiol, purchased from Sigma-Aldrich^®^ (St. Louis, MO, USA), and benzenethiol, from Merck^®^ (Kenilworth, NJ, USA). All solvents were commercially available and of reagent grade and were used without further purification. Melting points (mp) were determined on a SMP3 apparatus (Stuart Scientific, Staffordshire, United Kingdom) and were uncorrected. ^1^H-NMR spectra (400 MHz) were recorded on an AM-400 instrument (Bruker, Billerica, MA, USA) in deuterochloroform (CDCl_3_). ^13^C-NMR spectra were obtained in CDCl_3_ at 100 MHz. Peak assignment was confirmed by correlation with chemical structures in 2D experiments (HMBC, HSQC) performed by Valderrama et al. [35,36,37,38,39]. The assignments of chemical shifts are expressed in ppm downfield relative to tetramethylsilane (TMS, δ scale), and the coupling constants (*J*) are reported in Hertz. IR spectra were recorded on a Bruker Vector 22-FT spectrophotometer using KBr discs, and wavenumbers are reported in cm^−1^. HRMS were obtained on a model MAT 95XP spectrometer (Thermo Finnigan, Barkhausenstr, Germany), Silica gel (70–230 and 230–400 mesh) and TLC aluminum foil 60F254 Merck^®^ were used for preparative column chromatography and analytical TLC, respectively.

### 3.2. Chemical Synthesis

#### 3.2.1. Procedure for the Synthesis of Compound **1**

A suspension of 1-(2,5-dihydroxyphenyl)-propan-1-one 939 mg (5.65 mmol), 6-amino-1, 3-dimethyl-2,4(1*H*,3*H*)-pyrimidinedione 1068 mg (6.88 mmol), Ag_2_O 3794 mg (16.37 mmol), anhydrous MgSO_4_ 1963 mg (16.3 mmol), in dichloromethane (40 mL) was stirred at room temperature for 4 h. The mixture was filtered with Celite^®^ and washed with dichloromethane. The solvent was removed under reduced pressure and the crude of reaction was purified using 65 g of silica gel (230–400 mesh) using a mix of dichloromethane and ethyl acetate = 9:1. The resulting solution was concentrated to dryness under reduced pressure. The product, *6-ethyl-2,4-dimethylpyrimido[4,5-c]isoquinolin-1,3,7,10(2H,4H)-tetraone* (**1**): was obtained as a yellow solid; m.p. 167.6–167.9 °C; IR (KBr), ῡ = 1667 (C=O quinone), 1720 (C=O uracil) cm^−1^; ^1^H-NMR (CDCl_3_, 400 MHz) δ 7.11 (d, *J* = 10.3 Hz, 1H, H-9), 6.81 (d, *J* = 10.3 Hz, 1H, H-8), 3.76 (s, 3H, 2-*N*CH_3_), 3.47 (s, 3H, 4-*N*CH_3_), 3.40 (q, *J* = 7.3 Hz, 2H, 6-CH_2_CH_3_), 1.34 (t, *J* = 7.3 Hz, 3H, 6-CH_2_CH_3_); ^13^C-NMR (CDCl_3_, 100 MHz) δ 185.0 (1C, C-10), 183.9 (1C, C-7), 171.2 (1C, C-6), 159.0 (1C, C-4a), 152.9 (1C, C-1), 151.5 (1C, C-3), 146.6 (1C, C-10a), 138.7 (1C, C-8), 138.7 (1C, C-9), 121.2 (1C, C-6a), 105.4 (1C, C-10b), 32.0 (1C, 6-CH_2_CH_3_), 30.6 (1C, 2-*N*CH_3_), 29.5 (1C, 4-*N*CH_3_), 12.5 (1C, 6-CH_2_CH_3_); HRMS *m*/*z* 299.09070 (calcd for C_15_H_13_N_3_O_4_ [M]^+^, 299.09061); purified in column chromatography with dichloromethane: ethyl acetate = 9:1; Yield: 84%.

#### 3.2.2. General Procedure for the Synthesis of Compounds **2**–**17**

A solution of **1** (150 mg, 0.4909 mmol) and CeCl_3_.7H_2_O (5% mmol respect to **1**) in a mix of ethanol: dichloromethane = 1:1 (10 mL), was added dropwise slowly a solution of benzenethiol derivate (0.5 equiv.) in ethanol: dichloromethane = 1:1 (30 mL). The reaction mixture was stirred at room temperature for 16 h. The progress of the reaction was followed by thin-layer chromatography (TLC). The mixture was concentrated and the crude of reaction was purified using 65 g of silica gel (70–230 mesh) and a mix of dichloromethane, light petroleum and ethyl acetate than eluent in determinate proportions. The resulting solution was concentrated to dryness under reduced pressure.

*6-Ethyl-8-(phenylthio)-2,4-dimethylpyrimido[4,5-c]isoquinoline-1,3,7,10(2H,4H)-tetraone* (**2**): orange solid; m.p. 179.4–180.0 °C; IR (KBr), ῡ = 1658, 1676 (C=O quinone), 1720 (C=O uracil) cm^−1^; ^1^H-NMR (CDCl3, 400 MHz) δ 7.50–7.55 (m, 5H, SC_6_H_5_), 6.18 (s, 1H, H-9), 3.76 (s, 3H, 2-*N*CH_3_), 3.43 (s, 3H, 4-*N*CH_3_), 3.42 (q, *J* = 7.2 Hz, 2H, 6-CH_2_CH_3_), 1.37 (t, *J* = 7.3 Hz, 3H, 6-CH_2_CH_3_); ^13^C-NMR (CDCl3, 100 MHz) δ 181.8 (1C, C-10), 181.2 (1C, C-7), 171.2 (1C, C-6), 158.8 (1C, C-4a), 157.1 (1C, C-8), 153.1 (1C, C-1), 151.5 (1C, C-3), 147.7 (1C, C-10a), 136.1 (2C, C-2′ and C-6′), 131.1 (1C, C-4′), 130.9 (2C, C-3′ and C-5′), 128.3 (1C, C-9), 127.6 (1C, C-1′), 121.0 (1C, C-6a), 105.8 (1C, C-10b), 32.1 (1C, 6-CH_2_CH_3_), 30.6 (1C, 2-*N*CH_3_), 29.5 (1C, 4-*N*CH_3_), 12.6 (1C, 6-CH_2_CH_3_); HRMS *m*/*z* 407.09400 (calcd. for C_21_H_17_N_3_O_4_S [M]^+^, 407.09398); purified in column chromatography with dichloromethane: light petroleum: ethyl acetate = 9:8:1; Yield: 67%.

*6-Ethyl-8-((2*′*-methylphenyl)thio)-2,4-dimethylpyrimido[4,5-c]isoquinoline-1,3,7,10(2H,4H)-tetraone* (**3**): orange solid; m.p. 206.0–210.9 °C; IR (KBr), ῡ = 1660, 1688 (C=O quinone); 1730 (C=O uracil) cm^−1^; ^1^H-NMR (CDCl3, 400 MHz) δ 7.49 (d, *J* = 7.5 Hz, 1H, H-6′), 7.45–7.39 (m, 2H, H-5′ and H-3′), 7.30 (t, *J* = 6.8 Hz, 1H, H-4′), 6.01 (s, 1H, H-9), 3.75 (s, 3H, 2-*N*CH_3_), 3.43 (s, 3H, 4-*N*CH_3_), 3.42 (q, *J* = 6.6 Hz, 2H, 6-CH_2_CH_3_), 2.43 (s, 3H, 2′-CH_3_), 1.38 (t, *J* = 7.3 Hz, 3H, 6-CH_2_CH_3_); ^13^C-NMR (CDCl3, 100 MHz) δ 181.4 (1C, C-10), 181.1 (1C, C-7), 170.9 (1C, C-6), 158.6 (1C, C-4a), 155.6 (1C, C-8), 152.9 (1C, C-1), 151.2 (1C, C-3), 147.6 (1C, C-10a), 143.3 (1C, C-2′), 136.9 (1C, C-6′), 131.9 (1C, C-3′), 131.5 (1C, C-5′), 128.1 (1C, C-4′), 127.6 (1C, C-9), 126.5 (1C, C-1′), 120.9 (1C, C-6a), 105.7 (1C, C-10b), 31.9 (1C, 6-CH_2_CH_3_), 30.4 (1C, 2-*N*CH_3_), 29.2 (1C, 4-*N*CH_3_), 20.7 (1C, CH_3_-2′), 12.3 (1C, 6-CH_2_CH_3_); HRMS *m*/*z* 421.10957 (calcd. for C_22_H_19_N_3_O_4_S [M]^+^, 421.10963); purified in column chromatography with dichloromethane: light petroleum: ethyl acetate = 9:10:1; Yield: 72%.

*6-Ethyl-8-((2*′*-methoxyphenyl)thio)-2,4-dimethylpyrimido[4,5-c]isoquinoline-1,3,7,10(2H,4H)-tetraone* (**4**): orange solid, m.p. 172.3 (d) °C; IR (KBr), ῡ = 1660, 1688 (C=O quinone); 1727 (C=O uracil) cm^−1^; ^1^H-NMR (CDCl3, 400 MHz) δ 7.53–7.48 (m, 2H, H-4′ and H-6′), 7.02–7.07 (m, 2H, H-3′ and H-5′), 6.09 (s, 1H, H-9), 3.86 (s, 3H, 2′-CH_3_O), 3.74 (s, 3H, 2-*N*CH_3_), 3.42 (s, 3H, 4-*N*CH_3_), 3.41 (q, *J* = 7.2 Hz, 2H, 6-CH_2_CH_3_), 1.36 (t, *J* = 7.3 Hz, 3H, 6-CH_2_CH_3_); ^13^C-NMR (CDCl3, 100 MHz) δ 181.4 (1C, C-10), 181.2 (1C, C-7), 170.7 (1C, C-6), 160.1 (1C, C-2′), 158.6 (1C, C-4a), 154.8 (1C, C-8), 152.8 (1C, C-1), 151.2 (1C, C-3), 147.4 (1C,10a), 137.6 (1C, C-4′), 133.1 (1C, C-6′), 127.6 (1C, C-9), 122.1 (1C, C-5′), 120.9 (1C, C-6a), 114.6 (1C, C-1′), 112.1 (1C, C-3′), 105.5 (1C, C-10b), 56.2 (1C, 2′-OCH_3_), 31.8 (1C, 6-CH_2_CH_3_), 30.2 (1C, 2-*N*CH_3_), 29.1 (1C, 4-*N*CH_3_), 12.2 (1C, 6-CH_2_CH_3_); HRMS *m*/*z* 437.10450 (calcd. for C_22_H_19_N_3_O_5_S [M]^+^, 437.10454); purified in column chromatography with dichloromethane: light petroleum: ethyl acetate = 9:6:1; Yield: 72%.

*6-Ethyl-8-((2*′*-fluorobromophenyl)thio)-2,4-dimethylpyrimido[4,5-c]isoquinoline-1,3,7,10(2H,4H)-tetraone* (**5**): orange solid; m.p. 218.4 (d) °C; IR (KBr), ῡ = 1660, 1684 (C=O quinone); 1727 (C=O uracil) cm^−1^; ^1^H-NMR (CDCl3, 400 MHz) δ 7.60–7.54 (m, 2H, H-4′ and H-6′), 7.32–7.26 (m, 2H, H-3′ and H-5′), 6.13 (s, 1H, H-9), 3.76 (s, 3H, 2-*N*CH_3_), 3.44 (s, 3H, 4-*N*CH_3_), 3.43 (q, *J* = 7.2 Hz, 2H, 6-CH_2_CH_3_), 1.38 (t, *J* = 7.3 Hz, 3H, 6-CH_2_CH_3_); ^13^C-NMR (CDCl3, 100 MHz) δ 181.6 (1C, C-10), 181.1 (1C, C-7), 171.2 (1C, C-6), 163.1 (1C, d, *J* = 251.5, C-2′), 158.8 (1C, C-4a), 154.4 (1C, C-8), 153.2 (1C, C-1), 151.5 (1C, C-3), 147.6 (1C, C-10a), 137.9 (1C, C-5′), 133.9 (1C, d, *J* = 8.1, C-4′), 128.4 (1C, C-9), 126.3 (1C, d, *J* = 3.9, C-6′), 120.9 (1C, C-6a), 117.6 (1C, d, *J* = 22.3, C-3′), 114.9 (1C, d, *J* = 18.6, C-1′), 105.9 (1C, C-10b), 32.1 (1C, 6-CH_2_CH_3_), 30.6 (1C, 2-*N*CH_3_), 29.5 (1C, 4-*N*CH_3_), 12.5 (1C, 6-CH_2_CH_3_); HRMS *m*/*z* 425.08460 (calcd. for C_21_H_16_FN_3_O_4_S [M]^+^, 425.08455); purified in column chromatography with dichloromethane: light petroleum: ethyl acetate = 9:14:2; Yield: 87%.

*6-Ethyl-8-((2*′*-chlorophenyl)thio)-2,4-dimethylpyrimido[4,5-c]isoquinoline-1,3,7,10(2H,4H)-tetraone* (**6**): orange solid; m.p. 220.8 (d) °C; IR (KBr), ῡ = 1660, 1678 (C=O quinone); 1720 (C=O uracil) cm^−1^; ^1^H-NMR (CDCl3, 400 MHz) δ 7.60–7.64 (m, 2H, H-3′ and H-6′); 7.49 (t, *J* = 7.7 Hz, 1H, H-4′), 7.39 (t, *J* = 7.5 Hz, 1H, H-5′), 6.05 (s, 1H, H-9), 3.75 (s, 3H, 2-*N*CH_3_), 3.43 (s, 3H, 4-*N*CH_3_), 3.42 (q, *J* = 7.2 Hz, 2H, 6-CH_2_CH_3_), 1.37 (t, *J* = 7.3 Hz, 3H, 6-CH_2_CH_3_); ^13^C-NMR (CDCl3, 100 MHz) δ 181.6 (1C, C-10), 181.1 (1C, C-7), 171.2 (1C, C-6), 158.8 (1C, C-4a), 154.2 (1C, C-8), 153.2 (1C, C-1), 151.5 (1C, C-3), 147.6 (1C, C-10a), 140.3 (1C, C-2′), 138.4 (1C, C-6′), 132.9 (1C, C-4′), 131.6 (1C, C-3′), 128.9 (1C, C-5′), 128.3 (1C, C-9), 126.9 (1C, C-1′), 120.9 (1C, C-6a), 105.9 (1C, C-10b), 32.7 (1C, 6-CH_2_CH_3_), 30.2 (1C, 2-*N*CH_3_), 29.1 (1C, 4-*N*CH_3_), 12.1 (1C, 6-CH_2_CH_3_); HRMS *m*/*z* 441.05521 (calcd. for C_21_H_16_ClN_3_O_4_S [M]^+^, 441.05500); purified in column chromatography with dichloromethane: light petroleum: ethyl acetate = 9:14:2; Yield: 82%.

*6-Ethyl-8-((2*′*-bromophenyl)thio)-2,4-dimethylpyrimido[4,5-c]isoquinoline-1,3,7,10(2H,4H)-tetraone* (**7**): orange solid; m.p. 208.3 (d) °C; IR (KBr), ῡ = 1660; 1688 C=O (quinone); 1730 C=O (uracil) cm^−1^; ^1^H-NMR (CDCl3, 400 MHz) δ 7.79 (d, *J* = 7.8 Hz, 1H, H-3′), 7.65 (d, *J* = 7.5 Hz, 1H, H-6′), 7.44 (dt, *J* = 7.6 Hz, *J* =1.1 Hz, 1H, H-4′ or H-5′), 7.39 (dt, *J* = 7.6 Hz, *J* =1.1 Hz, 1H, H-5′ or H-4′), 6.05 (s, 1H, 9-H), 3.75 (s, 3H, 2-*N*CH_3_), 3.43 (s, 3H, 4-*N*CH_3_), 3.42 (q, *J* = 7.2 Hz, 2H, 6-CH_2_CH_3_), 1.39 (t, *J* = 7.3 Hz, 3H, 6-CH_2_CH_3_); ^13^C-NMR (CDCl3, 100 MHz) δ 181.2 (1C, C-10), 180.7 (1C, C-7), 170.9 (1C, C-6), 158.5 (1C, C-4a), 154.1 (1C, C-8), 152.9 (1C, C-1), 151.2 (1C, C-3), 147.3 (1C, C-10a), 138.0 (1C, C-6′), 134.7 (1C, C-3′), 132.5 (1C, C-4′), 130.8 (1C, C-2′), 129.3 (1C, C-5′), 128.9 (1C, C-1′), 127.9 (1C, C-9), 120.7 (1C, C-6a), 105.6 (1C, C-10b), 31.8 (1C, 6-CH_2_CH_3_), 30.3 (1C, 2-*N*CH_3_), 29.1 (1C, 4-*N*CH_3_), 12.2 (1C, 6-CH_2_CH_3_); HRMS *m*/*z* 485.00455 (calcd. for C_21_H_16_BrN_3_O_4_S [M]^+^, 485.00449); purified in column chromatography with dichloromethane: light petroleum: ethyl acetate = 9:12:1, Yield: 82%.

*6-Ethyl-8-((3′-methylphenyl)thio)-2,4-dimethylpyrimido[4,5-c]isoquinoline-1,3,7,10(2H,4H)-tetraone* (**8**)*:* orange solid; m.p. 162.0–163.0 °C; IR (KBr), ῡ =1561(C=O quinone), 1661; 1682 (C=O uracil) cm^−1^; ^1^H-NMR (CDCl3, 400 MHz) δ 7.40–7.36 (m, 1H, H-4′), 7.32–7.30 (m, 3H, H-2′, H-5′ and H-6′), 6.18 (s, 1H, H-9), 3.74 (s, 3H, 2-*N*CH_3_), 3.42 (s, 3H, 4-*N*CH_3_), 3.41 (q, *J* = 7.3 Hz, 2H, 6-CH_2_CH_3_), 2.40 (s, 3H, 3′-CH_3_), 1.36 (t, *J* = 7.3 Hz, 3H, 6-CH_2_CH_3_); ^13^C-NMR (CDCl3, 100 MHz) δ 181.5 (1C, C-10), 180.9 (1C, C-7), 170.8 (1C, C-6), 158.5 (1C, C-4a), 157.0 (1C, C-8), 152.8 (1C, C-1), 151.2 (1C, C-3), 147.4 (1C, C-10a), 140.6 (1C, C-3′), 136.2 (1C, C-2′), 132.7 (1C, C-6′), 131.6 (1C, C-5′), 130.3 (1C, C-4′), 128.0 (1C, C-9), 126.9 (1C, C-1′), 120.7 (1C, C-6a), 105.5 (1C, C-10b), 31.8 (1C, 6-CH_2_CH_3_), 30.3 (1C, 2-*N*CH_3_), 29.1 (1C, 4-*N*CH_3_), 21.4 (1C, 3′-CH_3_), 12.2 (1C, 6-CH_2_CH_3_); HRMS *m*/*z* 421.10960 (calcd. for C_22_H_19_N_3_O_4_S [M]^+^, 421.10963); purified in column chromatography with dichloromethane: light petroleum: ethyl acetate = 1:3:1; Yield: 47%.

*6-Ethyl-8-((3′-methoxyphenyl)thio)-2,4-dimethylpyrimido[4,5-c]isoquinoline-1,3,7,10(2H,4H)-tetraone* (**9**): orange solid; m.p. 179.5–180.5 °C; IR (KBr), ῡ = 1560, 1579 (C=O quinone), 1676 (C=O uracil) cm^−1^; ^1^H-NMR (CDCl3, 400 MHz) δ 7.40 (t, *J* = 7.7 Hz, 1H, H-5′), 7.10 (d, 1H, H-4′), 7.03 (d, 2H, H-2′ and H-6′), 6.21 (s, 1H, H-9), 3.83 (s, 3H, 3′-OCH_3_), 3.74 (s, 3H, 2-*N*CH_3_), 3.42 (s, 3H, 4-*N*CH_3_), 3.40 (q, *J* = 7.2 Hz, 2H, 6-CH_2_CH_3_), 1.36 (t, *J* = 7.2 Hz, 3H, 6-CH_2_CH_3_); ^13^C-NMR (CDCl3, 100 MHz) δ 181.4 (1C, C-10), 180.8 (1C, C-7), 170.7 (1C, C-6), 160.8 (1C, C-3′), 158.4 (1C, C-4a), 156.7 (1C, C-8), 152.8 (1C, C-1), 151.1 (1C, C-3), 147.4 (1C, C-10b), 131.3 (1C, C-5′), 128.2 (1C, C-9), 128.0 (1C, C-1′), 127.8 (1C, C-6′), 120.7 (1C, C-2′), 120.6 (1C, C-6a), 116.7 (1C, C-4′), 105.5 (1C, C-10b), 55.6 (1C, 3′-OCH_3_), 31.8 (1C, 6-CH_2_CH_3_), 30.3 (1C, 2-*N*CH_3_), 29.1 (1C, 4-*N*CH_3_), 12.2 (1C, 6-CH_2_CH_3_); HRMS *m*/*z* 437.10449 (calcd. for C_22_H_19_N_3_O_5_S [M]^+^, 437.10454); purified in column chromatography with dichloromethane: light petroleum: ethyl acetate = 3:3:1, Yield 66%. 

*6-Ethyl-8-((3′-fluorophenyl)thio)-2,4-dimethylpyrimido[4,5-c]isoquinoline-1,3,7,10(2H,4H)-tetraone* (**10**): orange solid; m.p. 170–171 °C; IR (KBr), ῡ = 1563 (C=O quinone); 1667; 1683 (C=O uracil) cm^−1^; ^1^H-NMR (CDCl3, 400 MHz) δ 7.51 (q, *J* = 7.5 Hz, 1H, H-5′), 7.35 (d, *J* = 7.6 Hz, 1H, H-2′), 7.30–7.23 (m, 2H, H-4′ and H-6′), 6.21 (s, 1H, H-9), 3.76 (s, 3H, 2-*N*CH_3_), 3.44 (s, 3H, 4-*N*CH_3_), 3.42 (q, *J* = 7.2 Hz, 2H, 6-CH_2_CH_3_), 1.37 (t, *J* = 7.3 Hz, 3H, 6-CH_2_CH_3_); ^13^C-NMR (CDCl3, 100 MHz) δ 181.3 (1C, C-10); 180.6 (1C, C-7), 170.9 (1C, C-6), 163.3 (1C, d, *J* = 251.9, C-3′), 158.4 (1C, C-4a), 155.9 (1C, C-8), 152.9 (1C, C-1), 151.1 (1C, C-3), 147.2 (1C, C-10b), 131.9 (1C, d, *J* = 8.2, C-5′), 131.6 (1C, d, *J* = 3.2, C-6′), 129.3 (1C, d, *J* = 7.6, C-1′), 128.1 (1C, C-9), 122.7 (1C, d, *J* = 22.1, C-2′), 120.5 (1C, C-6a), 118.16 (1C, d, *J* = 20.9, C-4′), 105.5 (1C, C-10a), 31.8 (1C, 6-CH_2_CH_3_), 30.3 (1C, 2-*N*CH_3_), 29.1 (1C, 4-*N*CH_3_), 12.2 (1C, 6-CH_2_CH_3_); HRMS *m*/*z* 425.08457 (calcd. for C_21_H_16_FN_3_O_4_S [M]^+^, 425.08455); purified in column chromatography with dichloromethane: light petroleum: ethyl acetate = 1:6:1, Yield 71%.

*6-Ethyl-8-((3′-chlorophenyl)thio)-2,4-dimethylpyrimido[4,5-c]isoquinoline-1,3,7,10(2H,4H)-tetraone* (**11**)*:* orange solid; m.p. 156.1–157.1 °C; IR (KBr), ῡ = 1558 (C=O quinone), 1662, 1681 (C=O uracil) cm^−1^; ^1^H-NMR (CDCl3, 400 MHz) δ 7.54 (s, 1H, H-2′), 7.50 (d, *J* = 7.1 Hz, 1H, H-4′), 7.47–7.42 (m, 2H, H-6′ and H-5′), 6.19 (s, 1H, H-9), 3.74 (s, 3H, 2-*N*CH_3_), 3.42 (s, 3H, 4-*N*CH_3_), 3.40 (q, *J* = 7.4 Hz, 2H, 6-CH_2_CH_3_), 1.36 (t, *J* = 7.3 Hz, 3H, 6-CH_2_CH_3_); ^13^C-NMR (CDCl3, 100 MHz) δ 181.3 (1C, C-10), 180.6 (1C, C-7), 170.9 (1C, C-6), 158.4 (1C, C-4a), 155.8 (1C, C-8), 152.9 (1C, C-1), 151.1 (1C, C-3), 147.2 (1C, C-10a), 136.1 (1C, C-3′), 135.5 (1C, C-2′), 133.9 (1C, C-5′), 131.5 (1C, C-4′), 131.1 (1C, C-6′), 129.1 (1C, C-1′), 128.2 (1C, C-9), 120.5 (1C, C-6a), 105.5 (1C, C-10b), 31.8 (1C, 6-CH_2_CH_3_), 30.3 (1C, 2-*N*CH_3_), 29.1 (1C, 4-*N*CH_3_), 12.2 (1C, 6-CH_2_CH_3_); HRMS *m*/*z* 441.05514 (calcd. for C_21_H_16_ClN_3_O_4_S [M]^+^, 441.05500); purified in column chromatography with dichloromethane: light petroleum: ethyl acetate = 1:3:1, Yield: 58%.

*6-Ethyl-8-((3′-bromophenyl)thio)-2,4-dimethylpyrimido[4,5-c]isoquinoline-1,3,7,10(2H,4H)-tetraone* (**12**): orange solid; m.p. 138.3–139.3°C; IR (KBr), ῡ = 1559 C=O (quinone); 1668 C=O (uracil) cm^−1^; ^1^H-NMR (CDCl3, 400 MHz) δ 7.69 (s, 1H, H-2′), 7.66 (d, *J* = 8.0 Hz, 1H, H-6′), 7.48 (d, *J* = 7.6 Hz, 1H, H-4′), 7.39 (t, *J* = 7.8 Hz, 1H, H-5′), 6.15 (s, 1H, H-9), 3.75 (s, 3H, 2-*N*CH_3_), 3.43 (s, 3H, 4-*N*CH_3_), 3.40 (q, *J* = 7.4 Hz, 2H, 6-CH_2_CH_3_), 1.36 (t, *J* = 7.3 Hz, 3H, 6-CH_2_CH_3_). ^13^C-NMR (CDCl3, 100 MHz) δ 181.3 (1C, C-10), 180.6 (1C, C-7), 170.9 (1C, C-6), 158.4 (1C, C-4a), 155.9 (1C, C-8), 152.9 (1C, C-1), 151.1 (1C, C-3), 147.2 (1C, C-10a), 138.3 (1C, C-2′), 134.4 (1C, C-4′), 134.0 (1C, C-6′), 131.8 (1C, C-5′), 129.5 (1C, C-3′), 128.2 (1C, C-9), 124.0 (1C, C-1′), 120.5 (1C, C-6a), 105.5 (1C, C-10b), 31.8 (1C, 6-CH_2_CH_3_), 30.3 (1C, 2-*N*CH_3_), 29.2 (1C, 4-*N*CH_3_), 12.2 (1C, 6-CH_2_CH_3_); HRMS *m*/*z* 485.00453 (calcd. for C_21_H_16_BrN_3_O_4_S [M]^+^, 485.00449); purified in column chromatography with dichloromethane: light petroleum: ethyl acetate = 1:8:1, Yield: 82%.

*6-Ethyl-8-((4′-methylphenyl)thio)-2,4-dimethylpyrimido[4,5-c]isoquinoline-1,3,7,10(2H,4H)-tetraone* (**13**): orange solid; m.p. 191.0–192.3 °C; IR (KBr), ῡ = 1662; 1687 (C=O quinone); 1726 (C=O uracil) cm^−1^; ^1^H-NMR (CDCl3, 400 MHz) δ 7.40 (d, *J* = 8.1 Hz, 2H, H-2′ and H-6′), 7.31 (d, *J* = 7.9 Hz, 2H, H-3′ and H-5′), 6.17 (s, 1H, H-9), 3.75 (s, 3H, 2-*N*CH_3_), 3.43 (s, 3H, 4-*N*CH_3_), 3.42 (q, *J* = 7.3 Hz, 2H, 6-CH_2_CH_3_), 2.43 (s, 3H, 4′-CH_3_), 1.37 (t, *J* = 7.3 Hz, 3H, 6-CH_2_CH_3_); ^13^C-NMR (CDCl3, 100 MHz) δ 181.8 (1C, C-10), 181.3 (1C, C-7), 171.1 (1C, C-6), 158.8 (1C, C-4a), 157.5 (1C, C-8), 153.5 (1C, C-1), 153.1 (1C, C-3), 147.8 (1C, C-10a), 141.6 (1C, C-4′), 136.0 (2C, C-2′ and C-6′), 131.6 (2C, C-3′ and C-5′), 128.2 (1C, C-9), 123.9 (1C, C-1′), 121.0 (1C, C-6a), 105.8 (1C, C-10b), 32.1 (1C, 6-CH_2_CH_3_), 30.6 (1C, 2-*N*CH_3_), 29.5 (1C, 4-*N*CH_3_), 21.8 (1C, 4′-CH_3_), 12.6 (1C, 6-CH_2_CH_3_); HRMS *m*/*z* 421.10954 (calcd. for C_22_H_19_N_3_O_4_S [M]^+^, 421.10963); purified in column chromatography with dichloromethane: light petroleum: ethyl acetate = 1:3:1, Yield: 88%.

*6-Ethyl-8-((4′-methoxyphenyl)thio)-2,4-dimethylpyrimido[4,5-c]isoquinoline-1,3,7,10(2H,4H)-tetraone* (**14**): red solid; m.p. 198.9–201.5 °C; IR (KBr), ῡ = 1662, 1689 (C=O quinone), 1726 (C=O uracil) cm^−1^; ^1^H-NMR (CDCl3, 400 MHz) δ 7.42 (d, *J* = 8.8 Hz, 2H, H-2′ and H-6′), 7.01 (d, *J* = 8.8 Hz, 2H, H-3′ and H-5′), 6.15 (s, 1H, H-9), 3.86 (s, 3H, 4′-OCH_3_), 3.75 (s, 3H, 2-*N*CH_3_), 3.43 (s, 3H, 4-*N*CH_3_), 3.40 (q, *J* = 7.3 Hz, 2H, 6-CH_2_CH_3_), 1.36 (t, *J* = 7.3 Hz, 3H, 6-CH_2_CH_3_); ^13^C-NMR (CDCl3, 100 MHz) δ 181.4 (1C, C-10), 181.1 (1C, C-7), 171.1 (1C, C-6), 162.0 (1C, C-4′), 158.8 (1C, C-4a), 157.9 (1C, C-8), 153.1 (1C, C-1), 151.5 (1C, C-3), 147.8 (1C, C-10a), 138.0 (2C, C-2′ and C-6′), 128.2 (1C, C-9), 121.0 (1C, C-6a), 117.6 (1C, C-1′), 116.5 (2C, C-3′ and C-5′), 105.8 (1C, C-10b), 56.0 (1C, 4′-OCH_3_), 32.1 (1C, 6-CH_2_CH_3_), 30.6 (1C, 2-*N*CH_3_), 29.5 (1C, 4-*N*CH_3_), 12.6 (1C, 6-CH_2_CH_3_); HRMS *m*/*z* 437.10454 (calcd. for C_22_H_19_N_3_O_5_S [M]^+^, 437.10454); purified in column chromatography with dichloromethane: light petroleum: ethyl acetate = 3:3:1; Yield: 82%.

*6-Ethyl-8-((4′-fluorophenyl)thio)-2,4-dimethylpyrimido[4,5-c]isoquinoline-1,3,7,10(2H,4H)-tetraone* (**15**)*:* orange solid; m.p. 194.9–195.4 °C; IR (KBr), ῡ = 1660; 1675 (C=O quinone); 1720 (C=O uracil) cm^−1^; ^1^H-NMR (CDCl3, 400 MHz) δ 7.50–7.47 (m, 2H, H-3′ and H-5′), 7.19–7.15 (m, 2H, H-2′ and H-6′), 6.16 (s, 1H, H-9), 3.77 (s, 3H, 2-*N*CH_3_), 3.39 (s, 3H, 4-*N*CH_3_), 3.37 (q, *J* = 7.3 Hz, 2H, 6-CH_2_CH_3_), 1.38 (t, *J* = 7.3 Hz, 3H, 6-CH_2_CH_3_); ^13^C-NMR (CDCl3, 100 MHz) δ 181.7 (1C, C-10), 181.1 (1C, C-7), 171.2 (1C, C-6), 164.6 (1C, d, *J* = 251, C-4′), 158.8 (1C, C-4a), 156.9 (1C, C-8), 153.2 (1C, C-1), 151.5 (1C, C-3), 147.6 (1C, C-10a), 138.3 (2C, d, *J* = 8, C-2′ and C-6′), 128.3 (1C, C-9), 122.9 (1C, C-1′), 120.9 (1C, C-6a), 118.2 (2C, d, *J* = 22, C-3′ and C-5′), 105.8 (1C, C-10b), 32.1 (1C, 6-CH_2_CH_3_), 30.6 (1C, 2-*N*CH_3_), 29.5 (1C, 4-*N*CH_3_), 12.5 (1C, 6-CH_2_CH_3_); HRMS *m*/*z* 425.08462 (calcd. for C_21_H_16_FN_3_O_4_S [M]^+^, 425.08455); purified in column chromatography with dichloromethane: light petroleum: ethyl acetate = 1:6:1, Yield: 61%.

*6-Ethyl-8-((4′-chlorophenyl)thio)-2,4-dimethylpyrimido[4,5-c]isoquinoline-1,3,7,10(2H,4H)-tetraone* (**16**)*:* orange solid; m.p. 196.5–198.3 °C; IR (KBr), ῡ = 1656; 1675 (C=O quinone); 1722 (C=O uracil) cm^−1^; ^1^H-NMR (CDCl3, 400 MHz) δ 7.51–7.46 (m, 4H, H-2′, H-3′, H-5′ and H-6′), 6.17 (s, 1H, H-9), 3.76 (s, 3H, 2-*N*CH_3_), 3.44 (s, 3H, 4-*N*CH_3_), 3.41 (q, *J* = 7.3 Hz, 2H, 6-CH_2_CH_3_), 1.37 (t, *J* = 7.3 Hz, 3H, 6-CH_2_CH_3_); ^13^C-NMR (CDCl3, 100 MHz) δ 181.6 (1C, C-10), 180.7 (1C, C-7), 171.2 (1C, C-6), 158.7 (1C, C-4a), 156.4 (1C, C-8), 153.1 (1C, C-1), 151.4 (1C, C-3), 147.6 (1C, C-10a), 137.8 (1C, C-4′), 137.3 (2C, C-2′ and C-6′), 131.1 (2C, C-3′ and C-5′), 128.4 (1C, C-9), 126.0 (1C, C-1′), 120.8 (1C, C-6a), 105.8 (1C, C-10b), 32.1 (1C, 6-CH_2_CH_3_), 30.6 (1C, 2-*N*CH_3_), 29.5 (1C, 4-*N*CH_3_), 12.5 (1C, 6-CH_2_CH_3_); HRMS *m*/*z* 441.05491 (calcd. for C_21_H_16_ClN_3_O_4_S [M]^+^, 441.05500); purified in column chromatography with dichloromethane: light petroleum: ethyl acetate = 1:3:1, Yield: 75%. 

*6-Ethyl-8-((4′-bromophenyl)thio)-2,4-dimethylpyrimido[4,5-c]isoquinoline-1,3,7,10(2H,4H)-tetraone* (**17**): yellow solid; m.p. 197.9–198.7 °C; IR (KBr), ῡ = 1660; 1677 C=O (quinone); 1722 C=O (uracil) cm^−1^; ^1^H-NMR (CDCl3, 400 MHz) δ 7.65 (d, *J* = 8.4 Hz, 2H, H-3′ and H-5′), 7.40 (d, *J* = 8.4 Hz, 2H, H-2′ and H-6′), 6.18 (s, 1H, H-9), 3.75 (s, 3H, 2-*N*CH_3_), 3.44 (s, 3H, 4-*N*CH_3_), 3.41 (q, *J* = 7.3 Hz, 2H, 6-CH_2_CH_3_), 1.36 (t, *J* = 7.3 Hz, 3H, 6-CH_2_CH_3_). ^13^C-NMR (CDCl3, 100 MHz) δ 181.6 (1C, C-10), 181.0 (1C, C-7), 171.2 (1C, C-6), 158.8 (1C, C-4a), 156.3 (1C, C-8), 153.2 (1C, C-1), 151.4 (1C, C-3), 147.5 (1C, C-10a), 137.6 (2C, C-2′ and C-6′), 134.1 (2C, C-3′ and C-5′), 128.4 (1C, C-9), 126.4 (1C, C-1′), 126.7 (1C, C-4′), 120.8 (1C, C-6a), 105.8 (1C, C-10b), 32.1 (1C, 6-CH_2_CH_3_), 30.6 (1C, 2-*N*CH_3_), 29.5 (1C, 4-*N*CH_3_), 12.5 (1C, 6-CH_2_CH_3_). HRMS *m*/*z* 485.00438 (calcd. for C_21_H_16_BrN_3_O_4_S [M]^+^, 485.00449), purified in column chromatography with dichloromethane: light petroleum: ethyl acetate = 1:8:1, Yield: 68%.

### 3.3. Crystalography

#### 3.3.1. Preparation of Single Crystals

Single crystals were grown by solvent evaporation at room temperature from the products of synthesis. Crystals suitable for X-ray diffraction studies were obtained from crystallization in saturated solutions: tetrahydropyran for **7** and benzene for **16**.

#### 3.3.2. Single Crystal X-ray Diffraction

Measured crystals were prepared under inert conditions and immersed in perfluoropolyether as a protective oil for manipulation. Suitable crystals were mounted on MiTeGen MicromountsTM, and these samples were used for data collection. Data were collected with a D8 Venture diffractometer CuKα, 298 K (Bruker, Karlsruhe, Germany). The data were processed with the APEX3 program [40] and corrected for absorption using SADABS [41]. The structures were resolved using direct methods [42], which revealed the position of all nonhydrogen atoms (Figures S1 and S2). These atoms were refined on F2 by a full-matrix least-squares procedure using anisotropic displacement parameters. All hydrogen atoms were located in difference Fourier maps and were included as fixed contributions riding on attached atoms with isotropic thermal displacement parameters 1.2 (C–H) or 1.5 (methyl) times those of the respective atom. CCDC 1573897 (compound **7**) and 1573896 (compound **16**) contain the crystallographic data listed in Table S2. These data can be obtained free of charge from the Cambridge Crystallographic Data Centre.

### 3.4. Evaluation of Antibacterial Activity and Cytotoxicity

#### 3.4.1. Bacterial Strains

As an initial screen for antibacterial activity, the compounds were tested against the following strains: methicillin-resistant *Staphylococcus aureus* (MRSA) ATCC^®^ 43300, methicillin-susceptible *Staphylococcus aureus* (MSSA) ATCC^®^ 29213, *Enterococcus faecalis* ATCC^®^ 29212, *Escherichia coli* ATCC^®^ 25922, and *Pseudomonas aeruginosa* ATCC^®^ 27853. The ISP provided the clinical isolates of MRSA [31] and VREF [32] used to calculate the MIC and MBC values of the most potent compounds. MRSA and VREF isolates that were resistant to at least three additional classes of antimicrobial agents were defined as multidrug resistant (MDR) [43]. All isolates were collected in 2014 and were obtained from various Chilean hospitals throughout the country (Table S1, Supplementary Material).

#### 3.4.2. Evaluation of Antibacterial Activity

##### Minimal Inhibitory Concentration Determination

MIC values were determined using a broth microdilution method, according to recommendations of the Clinical and Laboratory Standards Institute (CLSI) [44]. VAN and GEN were also tested against the strains and the results compared to the MIC ranges reported by the CLSI, as a quality control measure [23]. All compounds tested were dissolved in dimethyl sulfoxide (DMSO), to levels not exceeding 1% per well. MIC_50_ and MIC_90_ values were then calculated for the compounds observed to be the most active against the clinical isolates studied were also determined; MIC_50_ and MIC_90_ are the concentrations that inhibit 50% and 90% of the tested isolates, respectively. All assays were performed in triplicate and with *n* = 5.

##### Minimal bactericidal concentration determination

MBC values were then calculated for the compounds observed to be the most active against clinical isolates, following the recommendations of Pearson et al. [45], using an inoculum of 5 × 10^5^ CFU/mL. All wells with no visible growth observed in the microdilution assay were subcultured, extracting a 10-µL volume. Finally, MBC was determined based on the corresponding subcultured dilution plate in which the growth of microorganisms was less than 0.01% of the initial inoculum of 5 × 10^5^ CFU/mL. Compounds were classified as bactericides if the MBC/MIC ratio was equal to or less than 2 and as bacteriostatic if the ratio was greater than 2, according to Craig et al. [46] and Taylor et al. [47]. All assays were performed in triplicate and with *n* = 5.

#### 3.4.3. Cell Cultures

The cell lines used were obtained from the ATCC^®^ (Manassas, VA, USA) and included HeLa, human cervix adenocarcinoma (ATCC^®^ CCL-2); HTC-116, human colorectal carcinoma (ATCC^®^ CCL-247); SHSY-5Y, human neuroblastoma (ATCC^®^ CRL-2266); and Vero, monkey kidney fibroblast (ATCC^®^ CCL-81). Cells were grown in RPMI 1640 culture medium supplemented with 10% fetal bovine serum, 100 IU/mL penicillin, and 100 mg/mL streptomycin, in a humidified incubator in air with 5% CO_2_ at 37 °C.

#### 3.4.4. Evaluation of Cellular Toxicity

Cytotoxicity assays were performed using the MTS reduction method, as described previously [48]. Briefly, cancer cell lines were plated in a flat-bottom 96-well plate at 40,000 cells/mL. The cells were then incubated with test agents and VAN at escalating doses, ranging from 0 to 20 µg/mL, in triplicate in 200 µL of RPMI 1640 supplemented culture medium at 37 °C for 24 h. Five microliters of MTS was added to achieve a final concentration of 0.5 mg/mL and incubated at 37 °C for 2 h. Finally, formazan formation was measured in a multi-well reader at 540 nm (Stat Fax 4200, Awareness Technology, Inc., Palm City, FL, USA). The compounds were dissolved in DMSO, at levels not exceeding 1% per well. All assays were performed in triplicate and with *n* = 5. As a positive control for growth, wells containing only RPMI 140 medium were used. As a negative control of growth, H_2_O_2_ was used. As an additional negative control for growth, H_2_O + 1% DMSO was used. VAN and GEN were used as drug references.

### 3.5. Electrochemical Measuring

Cyclic voltammetry: Half-wave potentials (E_1/2_) were determined using a CHI 650 potentiostat (CH Instruments, Inc., 3700 Tennison Hill Drive Austin, TX, USA). Three-electrode cells with Ag^0^/AgCl or platinum wires were used as reference and auxiliary electrodes, respectively. The working electrode was prepared by polishing a glassy carbon electrode with 0.3 μm and 0.05 μm alumina and then washing with abundant deionized water. The compounds were dissolved to a concentration of 0.1 M with acetonitrile as a solvent and tetrabutylammonium perchlorate as a support electrolyte.

### 3.6. Determination of Theoretical Physicochemical Parameters

The lipophilic (LogP), and molar refractivity (MR) parameters were determined for each molecule using ChemDraw Ultra 12.0 (CambridgeSoft, Cambridge, MA, USA, www.cambridgesoft.com).

### 3.7. Statistical Analysis

The data were analyzed with one-way ANOVA and *t*-tests, with the criterion for statistical significance set at *p* < 0.05, using the GraphPad Prism 5.03 program (GraphPad Software, Inc., San Diego, California, USA, www.graphpad.com).

## 4. Conclusions

We synthesized 17 novel quinone compounds by employing a simple, fast, and economical two-step method with a yield of 47–88% yield. Antibacterial screening showed the compounds to have activity against Gram-positive MRSA, MSSA, and *E. faecalis* strains but not against Gram-negative *E.coli* and *P. aeruginosa*. 

Structural analysis showed that the presence of a thiophenolic ring is essential for activity. On the other hand, a study of the LogP, E_1/2_, and MR parameters showed that the addition of bulky substituents in the *ortho* position or the addition of lipophilic substituents in the *para* position improves the antibacterial activity. The results suggest that the antibacterial activity would be a function of more complex parameters that require extensive analysis. 

Compounds **7** and **16** were 128- and 64-fold more active against clinical isolates of VREF, respectively, than the drug VAN and did not affect the viability of HeLa, HTC-116, SHSY-5Y, or Vero cells in toxicity assays, demonstrating selectivity for prokaryotic cells. From a drug development point of view, the results of the new scaffold described herein support continuation of preclinical development to guide the synthesis of more potent agents to provide therapeutic options against infectious diseases provoked by Gram-positive MDR strains.

## 5. Patents

Chilean Patent Application number 201503780, PCT/CL2016/050080, EEUU 16/067,033; EPO 16880235.3; MX/a/2018/008192 titled: “Pyrimidine-Isoquinoline-Quinone Derived Compounds, their Salts, Isomers, Pharmaceutically Acceptable Tautomers; Pharmaceutical Composition; Preparation Procedure; and their Use in the Treatment of Bacterial and Multi-Resistant Bacterial Diseases”.

## Figures and Tables

**Table 1:** Antibacterial screening and physicochemical parameters for compounds **1**–**17**.

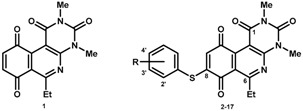										
N	R	MR (cm^3^/mol) of R	LogP	E_1/2_ (mV)	Mol wt (g/mol)	MIC (µg/mL)				
						MRSA	MSSA	*E. faecalis*	*E. coli*	*P. aeruginosa*
**1**	-	-	0.55	−0.539	299.28	>32	>32	>32	>32	>32
**2**	-H	0.80	0.74	−0.508	407.44	8	8	8	>32	>32
**3**	2′-Me	6.08	2.41	−0.549	421.47	32	32	>32	>32	>32
**4**	2′-OMe	7.24	1.80	−0.568	437.47	2	4	4	>32	>32
**5**	2′-F	1.64	2.09	−0.564	425.43	>32	>32	>32	>32	>32
**6**	2′-Cl	5.93	2.49	−0.623	441.89	>32	>32	>32	>32	>32
**7**	2′-Br	9.06	2.76	−0.604	486.34	1	4	2	>32	>32
**8**	3′-Me	6.08	2.41	−0.510	421.47	4	4	4	>32	>32
**9**	3′-OMe	7.24	1.80	−0.551	437.47	4	8	4	>32	>32
**10**	3′-F	1.64	2.09	−0.427	425.43	4	4	8	>32	>32
**11**	3′-Cl	5.93	2.49	−0.374	441.89	2	32	4	>32	>32
**12**	3′-Br	9.06	2.76	−0.443	486.34	2	32	4	>32	>32
**13**	4′-Me	6.08	2.41	−0.519	421.47	4	4	16	>32	>32
**14**	4′-OMe	7.24	1.80	−0.520	437.47	16	16	16	>32	>32
**15**	4′-F	1.64	2.09	−0.484	425.43	8	8	8	>32	>32
**16**	4′-Cl	5.93	2.49	−0.494	441.89	4	4	4	>32	>32
**17**	4′-Br	9.06	2.76	−0.501	486.34	4	8	8	>32	>32
	VAN ^a^					1	1	1	-	-
	GEN ^b^					-	-	-	0.5	1

^a^ Vancomycin, control quality for Gram-positive ATCC^®^ strains are 0.5–2 µg/mL against MRSA and MSSA; 1–4 µg/mL against E. faecalis according to CLSI [23]. ^b^ Gentamicin, control quality for Gram-negative ATCC^®^ strains are 0.25–1 µg/mL against E. coli and 0.25–2 against P. aeruginosa according to CLSI [23].

**Table 2:** Summary of antibacterial activity of compounds **7**, **16** and vancomycin against MDR *S. aureus*. MIC and MBC (µg/mL).

Compound	Isolates	MIC ^[b]^ Range	MIC_50_	MIC_90_	GM ^[c]^ MIC	MBC ^[d]^ Range	MBC_50_	MBC_90_	GM ^[c]^ MBC	MBC_50_/MIC_50_	MBC_90_/MIC_90_
**7**	32 ^[a]^	4–1	2	2	2.3	8–2	4	4	2.89	2	2
**16**	29 ^[a]^	4–1	2	4	2.11	4–1	2	4	3	1	1
VAN	45	2–1	1	1	1.13	ND	ND	ND	ND	-	-

^[a]^ isolates with MIC > 32 were not considered in the analysis; ^[b]^ MIC Minimum Inhibitory Concentration (µg/mL); ^[c]^ geometric mean; ^[d]^: Minimum Bactericidal Concentration (µg/mL); ND: Not determined. In triplicate and *n* = 5.

**Table 3:** Summary of antibacterial activity of compounds **7**, **16** and vancomycin against MDR *Enterococcus* sp. MIC and MBC (µg/mL).

Compound	Isolates	MIC ^[b]^ Range	MIC_50_	MIC_90_	GM ^[c]^_MIC_	MBC ^[d]^ Range	MBC_50_	MBC_90_	GM ^[c]^ MBC	MBC_50_/MIC_50_	MBC_90_/MIC_90_
**7**	44	4–2	2	4	2.51	8–2	4	4	3.42	2	1
**16**	41 ^[a]^	8–2	4	4	3.13	8–2	4	8	4	1	2
VAN	44	512–128	256	512	256.3	ND	ND	ND	ND	-	-

^[a]^ isolates with MIC > 32 were not considered in the analysis; ^[b]^ MIC Minimum Inhibitory Concentration (µg/mL); ^[c]^ geometric mean; ^[d]^ Minimum Bactericidal Concentration (µg/mL); ND: Not determined. In triplicate and *n* = 5.

## Supplementary Materials

The following are available online at http://www.mdpi.com/1420-3049/23/7/1776/s1, Table S1. Categorization of isolates by specimen type and multi drug-resistant patterns, Table S2. Crystal data and refinement details for compounds **7** and **16**, Figure S1. Molecular structure of **7** showing the atom-labelling scheme. Displacement ellipsoids are drawn at the 50% probability level. Figure S2. Molecular structure of 16 showing the atom-labelling scheme. Displacement ellipsoids are drawn at the 50% probability level.
