# Supplementary material for: A New Kind of Quinonic-Antibiotic Useful Against Multidrug-Resistant S. aureus and E. faecium Infections
Source: Molecules. 2018 Jul 19;23(7):1776. doi: 10.3390/molecules23071776 (PMC6100241; doi:10.3390/molecules23071776)
Supplement: Supplementary file 1 [file molecules-23-01776-s001.pdf]

A New Kind of Quinonic-Antibiotic Useful Against Multidrug-Resistant *S. aureus* and *E. faecium* Infections

Javier Campanini-Salinas [1,2 †], Juan Andrades-Lagos [1 †], Gerardo Gonzalez Rocha [3], Duane Choquesillo-Lazarte [4], Soledad Bollo Dragnic [5], Mario Faúndez [6], Pedro Alarcón [7], Francisco Silva [8], Roberto Vidal [9], Edison Salas-Huenuleo [10], Marcelo Kogan [10], Jaime Mella [11,12], Gonzalo Recabarren Gajardo [13] and David Vásquez-Velásquez [1,*]

1 Drug Development Laboratory, Faculty of Chemical and Pharmaceutical, Sciences, Universidad de Chile, Sergio Livingstone 1007, Santiago 8380492, Chile; javier.campanini@uss.cl (J.C.-S.); jandrades@ug.uchile.cl (J.A.-L.)
2 Facultad de Medicina y Ciencia, Universidad San Sebastián, Lago, Panguipulli 1390, Puerto Montt 5501842, Chile
3 Laboratorio de Investigación en Agentes Antibacterianos (LIAA), Departamento de Microbiología, Facultad de Ciencias Biológicas, Universidad de Concepción, Concepción 4070386, Chile; ggonzal@udec.cl
4 Laboratorio de Estudios Cristalográficos, IACT (CSIC-UGR), Av. de las Palmeras 4, Armilla (Granada) 18100, Spain; duane.choquesillo@csic.es
5 Bioelectrochemistry Laboratory, Faculty of Chemical and Pharmaceutical, Sciences, Universidad de Chile, Santiago 8380492, Chile; sbollo@ciq.uchile.cl
6 Molecular Pharmacology and Toxicology Laboratory, Pharmacy Department, Faculty of Chemistry, Pontificia Universidad Católica de Chile; Santiago 7820436, Chile; mfaundeza@uc.cl
7 Agents of bacterial meningitis laboratory, Instituto de Salud Pública de Chile, Santiago 7780050, Chile; palarcon@ispch.cl
8 Microbiology Unit, Clinical Laboratory, Clinical Hospital University of Chile; Santiago 8380456, Chile fsilva@hcuch.cl
9 Antibiotics Laboratory, Microbiology Program, Biomedical Sciences Institute, Faculty of Medicine, Universidad de Chile, Santiago 8380453, Chile; rvidal@uchile.cl
10 Nanobiotechnology and Nanotoxicology Laboratory, Faculty of Chemical and Pharmaceutical Sciences, Universidad de Chile, Santiago 8380494, Chile; edison.salash@gmail.com (E.S.-H.); mkogan@ciq.uchile.cl (M.K.)
11 Institute of Chemistry and Biochemistry, Faculty of Sciences, Universidad de Valparaíso, Playa Ancha, Valparaíso 2360102, Chile; jaime.mella@uv.cl
12 Escuela de Quimica y Farmacia, Facultad de Medicina, Universidad Andres Bello, Quillota 980, Viña del Mar 2531015, Chile
13 Laboratory of Synthesis of Bioactive Heterocycles, Pharmacy Department, Faculty of Chemistry, Pontificia Universidad Católica de Chile; Santiago 7820436, Chile; grecabarren@uc.cl
† These authors contributed equally to this work.
* Correspondence: dvasquez@ciq.uchile.cl; Tel.: +56-02-2978-2887

Table S1. Categorization of isolates by specimen type and multi drug-resistant patterns

Species	Specimen type	Total no. of isolates	No. (%) MDR
Staphylococcus aureus (MR*SA*)	Wound	10	4 (40)
	Tracheal aspirate	10	7 (70)
	Blood	20	20 (100)
	Others	5	4 (80)
Enterococcus faecium (VRE)	Urine	10	10 (100)
	Peritoneal fluid	10	10 (100)
	Wound	10	10 (100)
	Blood	10	9 (90)
	Others	4	4 (100)

Table S2. Crystal data and refinement details for compounds **7** and **16**.

	7	16
Empirical formula	$C_{21}H_{16}BrN_3O_4S$	$C_{21}H_{16}ClN_3O_4S$
Formula weight	486.34	441.88
Crystal system	Orthorhombic	Orthorhombic
Space group	Pbcn	Fdd2
Unit cell dimensions [Å, º]	a = 25.210(2)	a = 31.777(2)
	b = 7.6818(5)	b = 55.879(6)
	c = 21.1932(13)	c = 4.4937(3)
	α= 90	α= 90
	β= 90	β= 90
	γ = 90	γ = 90
Volume [Å^3]	4104.3(5)	7979.3(11)
Z	8	16
$\rho_{calcd.}$ [g cm^{-3}]	1.574	1.471
Absorption coeff. [mm^{-1}]	3.977	2.976
F(000)	1968	3648
θ range for data collection [º]	4.172 to 66.625	3.163 to 66.956
Reflections collected	25987	25076
Independent reflections	3624 [R_{int} = 0.0946]	3552 [R_{int} = 0.0458]
Completeness to θ	66.63º, 99.8 %	66,96º, 99.9 %
Max. and min. transmission	0.7528 and 0.5575	0.7528 and 0.5067
Data / restraints / parameters	3624 / 0 / 274	3552 / 1 / 274
Goodness-of-fit on F^2	1.045	1.057
Final R indices [F^2>2σ(F^2)]	R_1 = 0.0587, wR_2 = 0.1376	R_1 = 0.0385, wR_2 = 0.0898
R indices (all data)	R_1 = 0.1090, wR_2 = 0.1682	R_1 = 0.0485, wR_2 = 0.0981

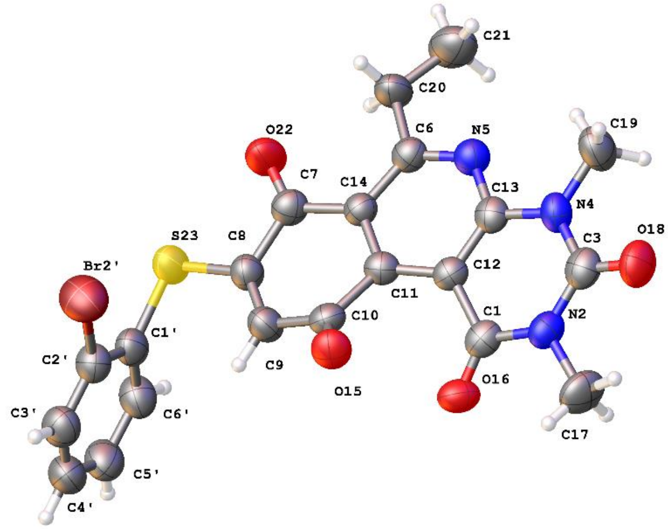

Figure S1. Molecular structure of **7** showing the atom-labelling scheme. Displacement ellipsoids are drawn at the 50 % probability level.

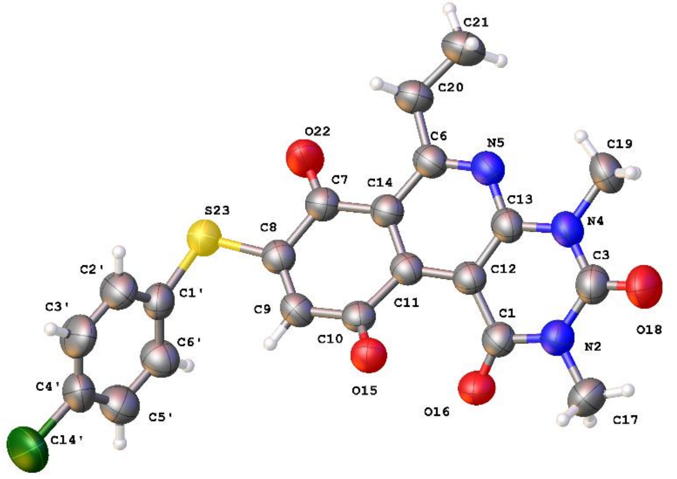

Figure S2. Molecular structure of **16** showing the atom-labelling scheme. Displacement ellipsoids are drawn at the 50 % probability level.
